# First case of *Trichophyton indotineae* in Brazil: clinical and mycological criteria and genetic identification of terbinafine resistance^☆^

**DOI:** 10.1016/j.abd.2025.01.001

**Published:** 2025-02-24

**Authors:** John Verrinder Veasey, Renata Diniz Jacques Gonçalves, Guilherme Camargo Julio Valinoto, Gustavo de Sá Menezes Carvalho, Giovanna Azevedo Celestrino, Ana Paula Carvalho Reis, Ana Paula Cordeiro Lima, Antonio Charlys da Costa, Marcia de Souza Carvalho Melhem, Gil Benard, Maria Gloria Teixeira Sousa

**Affiliations:** a Dermatology Clinic, Hospital da Santa Casa de São Paulo, São Paulo, SP, Brazil; b Discipline of Dermatology, Faculty of Medical Sciences, Santa Casa de São Paulo, São Paulo, SP, Brazil; c Private Practice, Piracicaba, SP, Brazil; d Laboratory of Medical Mycology LIM-53, Division of Clinical Dermatology, Instituto de Medicina Tropical, Hospital das Clínicas, Faculty of Medicine, Universidade de São Paulo, São Paulo, SP, Brazil; e Department of Pathology, Faculty of Medicine, Universidade de São Paulo, São Paulo, SP, Brazil; f Laboratory of Virology (LIM 52), Instituto de Medicina Tropical, Universidade de São Paulo, São Paulo, SP, Brazil

*Dear Editor,*

Terbinafine-resistant dermatophytes are currently a global health problem, particularly *Trichophyton indotineae*. Terbinafine, a potent antifungal agent against dermatophytes, inhibits the enzyme squalene epoxidase (SQLE), restricting fungal growth by interfering with ergosterol biosynthesis; point mutations in the *SQLE* genes are the main cause of antifungal resistance.^1^ *T. indotineae*, formerly known as *Trichophyton mentagrophytes* variety VIII, frequently shows mutations in the *SQLE* gene.^2^, ^3^ The present report describes the first case of a patient diagnosed in Brazil with dermatophytosis caused by terbinafine-resistant *T. indotineae*.

A 40-year-old male individual, without comorbidities, originally from Brazil and living in London, reported pruritic erythematous-desquamative lesions on the lower limbs and buttocks that began in January 2024 (Fig. 1). He reported frequent short trips during the second half of 2023; to Austria, Slovakia, Hungary, and Poland in August, and to Scotland and Turkey in November and December. He sought dermatological care in Piracicaba (state of São Paulo, Brazil), where direct mycological examination revealed the presence of hyaline septate hyphae, and the culture using Sabouraud agar and Mycosel® showed growth of *T. mentagrophytes*. He was diagnosed with dermatophytosis and in March 2024 he was prescribed 500 mg/day of terbinafine for 14 days.

**Figure 1:**
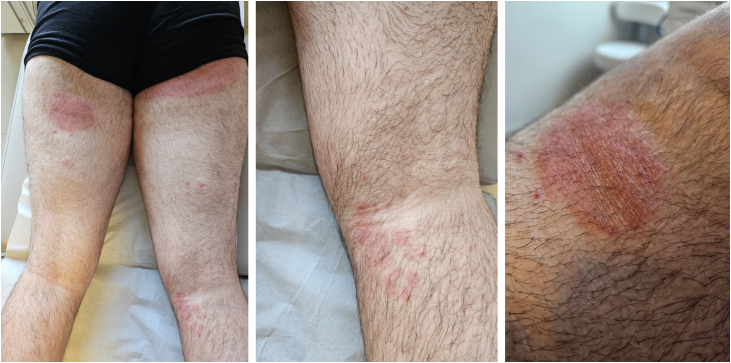
Clinical aspect of dermatophytosis lesions caused by *Trichophyton indotineae* affecting the posterior region of the lower limbs.

Although there was no clinical improvement, the patient returned to London; in May 2024 he came back presenting lesions in the same locations (Fig. 2). On this occasion, itraconazole 200 mg/day was prescribed for 14 days, with complete clinical remission. However, the patient developed recurrence after treatment was discontinued, and fluconazole 150 mg/day was prescribed for seven days, which proved ineffective. With a new cycle of treatment using itraconazole at the same dosage, the patient showed the same result: good initial response followed by recurrence four days after treatment was discontinued. A skin scraping specimen was collected for new mycological analysis, and treatment with itraconazole was prescribed again. The condition improved, the patient returned to England and once again was lost to dermatological follow-up.

**Figure 2:**
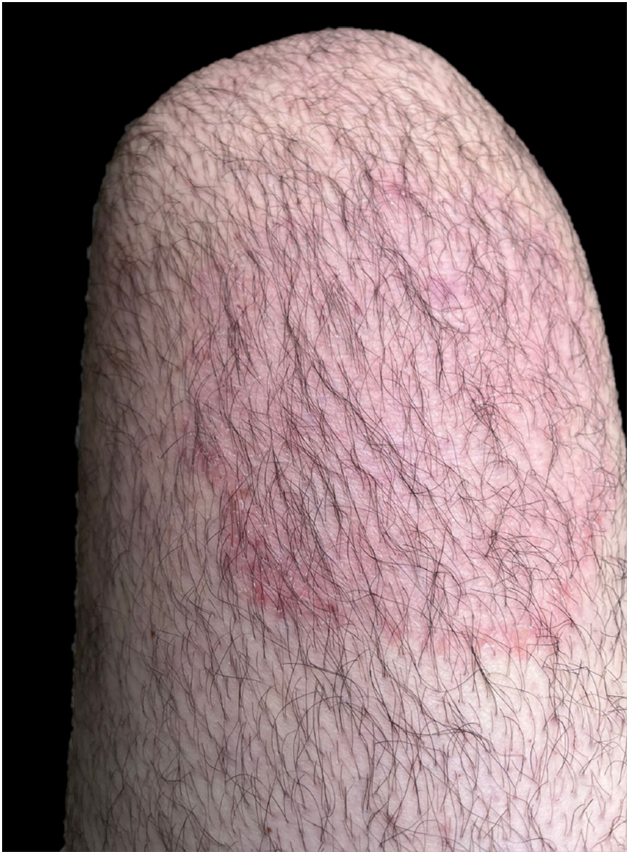
Clinical aspect of dermatophytosis recurrence caused by *Trichophyton indotineae* affecting the anterior surface of the right thigh.

In this scenario of (i) disseminated dermatophytosis refractory to terbinafine but susceptible to itraconazole, (ii) microbiological evidence suggestive of the *T. mentagrophytes*/*T. interdigitale* species complex, and (iii) history of frequent international travel, it was strongly suggested that this was a case of *T. indotineae*. This suspicion was confirmed using the material from the second collection, identifying the isolate as *T. indotineae* resistant to terbinafine and fluconazole through analysis of mycological exams (Fig. 3) associated with DNA sequencing of the internal transcribed spacer (ITS) region of ribosomal DNA. The *SQLE* gene was also amplified and sequenced using the described primers.^4^ The sequences were deposited in GenBank under access numbers PQ634380 (*T. indotineae*) and PQ655447 (*SQLE*). The sequences used are shown in Fig. 4. In addition, an antifungal susceptibility test for terbinafine, fluconazole, and itraconazole was performed using the *in vitro* broth microdilution reference method described by EUCAST (E.DEF 9.4).

**Figure 3:**
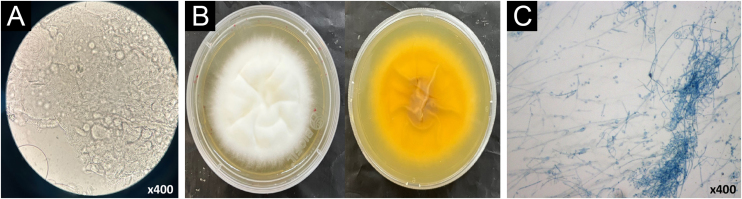
Mycological examinations of *Trichophyton indotineae*. (A) Direct microscopic examination (with 10% KOH) under optical microscopy (×400) showing branched septate hyaline hyphae and arthroconidia. (B) Macromorphology of fungal culture in Sabouraud medium showing velvety white front and light yellow pigment on the back. (C) Micromorphology under optical microscopy (×400), stained with lactophenol blue, showing the presence of numerous pyriform and clavate microconidia and septate, spindle-shaped macroconidia.

**Figure 4:**
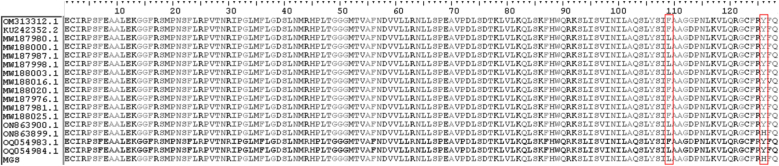
*SQLE* gene alignment under CLUSTAL multiple sequence alignment in BioEdit. The amino acid sequences of *SQLE* from the *T. indotineae* isolate IMT-1778 (MGS) were compared with the reference sequence of the *T. mentagrophytes* strain TIMM2789 (GenBank acc. number KU242352.) and the *T. interdigitale* isolate DK-Tinterdig-WT (GenBank acc. number OM313312.1), as well as *SQLE* sequences of terbinafine-resistant *T. indotineae* strains (GenBank acc. numbers MW187976, MW187980, MW187981, MW187987, MW187998, MW188000, MW188003, MW188016, MW188020, MW188025, ON863900, ON863899, OQ054983 and OQ054984). The amino acid substitutions that were found to be different in IMT-1778-MGS isolate are depicted, and their positions are shown in red boxes.

The assessed isolate is resistant to terbinafine and fluconazole, with minimum inhibitory concentration (MIC) values ​​of ≥16 µg/mL (upper limit value) and 8 µg/mL, respectively; and susceptible to itraconazole (MIC value of 0.064 µg/mL). Sequencing results revealed two terbinafine resistance mutations (Phe^397^Leu and Thr^414^His).

In the last decade, *T. indotineae* has caused large outbreaks of severe and difficult-to-treat infections worldwide. Lesions may be atypical with multiple morphologies, including concentric erythematous, desquamative, papulosquamous and pustular plaques, in addition to conditions modified by the use of topical corticosteroids.^5^

Cases of terbinafine-resistant *T. indotineae* described are often introduced by immigrants from endemic countries.^5^, ^6^ The high rate of inter-human transmission is a strong contributor to its spread, where familial cases account for about 50% of patients, and sharing of fomites is a common denominator.^5^, ^6^, ^7^, ^8^ However, few cases have been reported to date, mainly due to misidentification and underreporting.^6^ This may be the scenario in Brazil, where terbinafine-resistant dermatophytosis may be overlooked, since the etiological identification of dermatophytes remains a challenge, as DNA sequencing is not routinely used in the diagnosis of superficial mycoses.

The emergence of terbinafine-resistant *T. indotineae* is noteworthy, considering its frequency of up to 75% compared to 44% for *T. rubrum*.^9^, ^10^ This phenomenon may be linked to (i) inappropriate use of antibiotics, antifungals, and corticosteroids; (ii) climate change and indiscriminate use of pesticides; and (iii) the return of intense migratory movements seen after the COVID-19 pandemic.^7^, ^8^

In summary, the present case is the first report of dermatophytosis caused by *T. indotineae* in Brazil, with the typical evolution of therapeutic resistance to several antifungals and terbinafine resistance associated with mutations in the *SQLE* gene. Phenotypic and genotypic characterizations were essential for adequate diagnosis and therapeutic choice, but terbinafine resistance complicates treatment options and highlights the need for better surveillance, prevention strategies, and alternative therapeutic approaches.

## Financial support

None declared.

## Authors’ contributions

John Verrinder Veasey: Design and planning of the study; collection of data, or analysis and interpretation of data; drafting and editing of the manuscript or critical review of important intellectual content; effective participation in research orientation; intellectual participation in the propaedeutic and/or therapeutic conduct of the studied cases; critical review of the literature; approval of the final version of the manuscript.

Renata Diniz Jacques Gonçalves: Collection, analysis and interpretation of data; intellectual participation in the propaedeutic and/or therapeutic conduct of the studied cases; approval of the final version of the manuscript.

Guilherme Camargo Julio Valinoto: Approval of the final version of the manuscript; critical review of the literature.

Gustavo de Sá Menezes Carvalho: Approval of the final version of the manuscript; critical review of the literature.

Giovanna Azevedo Celestrino: Collection of data, or analysis and interpretation of data; critical review of the literature.

Ana Paula Carvalho Reis: Collection of data, or analysis and interpretation of data; critical review of the literature.

Ana Paula Cordeiro Lima: Collection of data, or analysis and interpretation of data; critical review of the literature.

Antonio Charlys da Costa: Collection of data, or analysis and interpretation of data; critical review of the literature.

Marcia de Souza Carvalho Melhem: Collection of data, or analysis and interpretation of data; critical review of the literature.

Gil Benard: Collection of data, or analysis and interpretation of data; critical review of the literature.

Maria Gloria Teixeira Sousa: Design and planning of the study; collection of data, or analysis and interpretation of data; drafting and editing of the manuscript or critical review of important intellectual content; effective participation in research orientation; critical review of the literature; approval of the final version of the manuscript.

## Conflicts of interest

None declared.
